# Canopy dieback and recovery in Australian native forests following extreme drought

**DOI:** 10.1038/s41598-022-24833-y

**Published:** 2022-12-14

**Authors:** Adriano Losso, Anthea Challis, Alice Gauthey, Rachael H. Nolan, Samuel Hislop, Adam Roff, Matthias M. Boer, Mingkai Jiang, Belinda E. Medlyn, Brendan Choat

**Affiliations:** 1 Hawkesbury Institute for the Environment, Western Sydney University, Locked Bag 1797, Penrith, NSW 2751 Australia; 2 Department of Botany, University of Innsbruck, Sternwartestraße 15, 6020 Innsbruck, Austria; 3 Plant Ecology Research Laboratory PERL, Ecole Polytechnique Fédérale de Lausanne EPFL, 1015 Lausanne, Switzerland; 4 Swiss Federal Institute for Forest, Snow and Landscape Research WSL, Zürcherstrasse 111, 8903 Birmensdorf, Switzerland; 5 NSW Bushfire Risk Management Research Hub, Wollongong, NSW Australia; 6 Forest Science, NSW Department of Primary Industries, Parramatta, NSW 2150 Australia; 7 Department of Planning, Industry and Environment, Remote Sensing and Landscape Science, 26 Honeysuckle Drive, Newcastle, NSW 2302 Australia; 8 College of Life Sciences, Zhejiang University, 866 Yuhangtang Rd, Hangzhou, Zhejiang China

**Keywords:** Plant ecology, Plant physiology, Drought

## Abstract

In 2019, south-eastern Australia experienced its driest and hottest year on record, resulting in massive canopy dieback events in eucalypt dominated forests. A subsequent period of high precipitation in 2020 provided a rare opportunity to quantify the impacts of extreme drought and consequent recovery. We quantified canopy health and hydraulic impairment (native percent loss of hydraulic conductivity, PLC) of 18 native tree species growing at 15 sites that were heavily impacted by the drought both during and 8–10 months after the drought. Most species exhibited high PLC during drought (PLC:65.1 ± 3.3%), with no clear patterns across sites or species. Heavily impaired trees (PLC > 70%) showed extensive canopy browning. In the post-drought period, most surviving trees exhibited hydraulic recovery (PLC:26.1 ± 5.1%), although PLC remained high in some trees (50–70%). Regained hydraulic function (PLC < 50%) corresponded to decreased canopy browning indicating improved tree health. Similar drought (37.1 ± 4.2%) and post-drought (35.1 ± 4.4%) percentages of basal area with dead canopy suggested that trees with severely compromised canopies immediately after drought were not able to recover. This dataset provides insights into the impacts of severe natural drought on the health of mature trees, where hydraulic failure is a major contributor in canopy dieback and tree mortality during extreme drought events.

## Introduction

The global increase in intense drought events is responsible for widespread forest decline and tree dieback, highlighting the importance of understanding and predicting the vulnerability of trees to more frequent extreme drought events expected in the future^1–3^. In forest ecosystems, the main consequences of extreme drought are increased canopy disturbance along with incomplete and lagged growth recovery^4,5^. Thus, it is of critical importance to understand when trees die after a drought event and the time taken for recovery of surviving trees (i.e., drought-legacy effect)^3,6^.

A range of factors, both biotic and abiotic, might ultimately contribute to the death of an individual tree during or following drought^4^, but recent studies emphasize the role of two interdependent mechanisms, hydraulic failure (i.e., a catastrophic level of xylem embolism, usually with native percent loss of hydraulic conductivity > 80%) and carbon starvation (i.e., prolonged stomatal closure during drought unbalances carbohydrate demand and supply and may lead to an inability to meet osmotic, metabolic and defensive carbon requirements), as the main causes for drought-induced mortality^7–10^. Drought-induced forest decline is particularly hard to study in mature trees and few studies have quantified the physiological response to extreme drought in the field and under natural conditions^1,11,12^. Predicting the timing of an extreme drought event and ensuring the availability of sufficient resources to perform physiological measurements is difficult, making these types of studies rare and challenging to plan. Therefore, field data providing insights into the physiological causes of mortality are extremely valuable and essential for validating model frameworks developed through manipulative experiments under controlled conditions^13,14^.

Most direct assessments of tree hydraulic vulnerability to drought are derived from laboratory and/or greenhouse experiments that artificially induce drought stress on either saplings or excised branches^15–18^. The few studies that provided direct evidence of hydraulic impairment (i.e., any level of xylem embolism) after natural drought are scattered across the globe and include data from a karstic woodland in north-east Italy^19^, a chaparral shrubland in California^20^, central European forests^1,21–23^, and eucalypt forests in eastern Australia^24^. All these studies have reported pronounced loss of hydraulic conductivity as well as different extents of leaf discoloration and premature leaf shedding. Specifically, Nardini et al.^19^ and Nolan et al.^24^ found hydraulic failure to be strongly associated with canopy dieback during drought. The recent study by Nolan et al.^24^ investigated the role of hydraulic failure and tree size on canopy dieback after the 2019 drought in three eucalypt tree species growing in one region in eastern Australia, and reported loss of hydraulic conductivity between 78 and 100% in trees with extensive canopy dieback. There are even fewer field data available documenting the recovery of trees in the long-term following dieback^4,25,26^. Studies examining physiological recovery from drought stress over the short term in smaller potted plants under controlled conditions provide conflicting evidence. While some studies undertaken with young plants in pots indicate a strong capacity for rapid recovery in hydraulic capacity by embolism repair^27,28^, others have suggested that embolism repair after drought is uncommon^29–31^. This lack of data represents a key knowledge gap in understanding of mechanisms governing recovery and potential legacy effects that persist for years after drought events.

**Figure 1:**
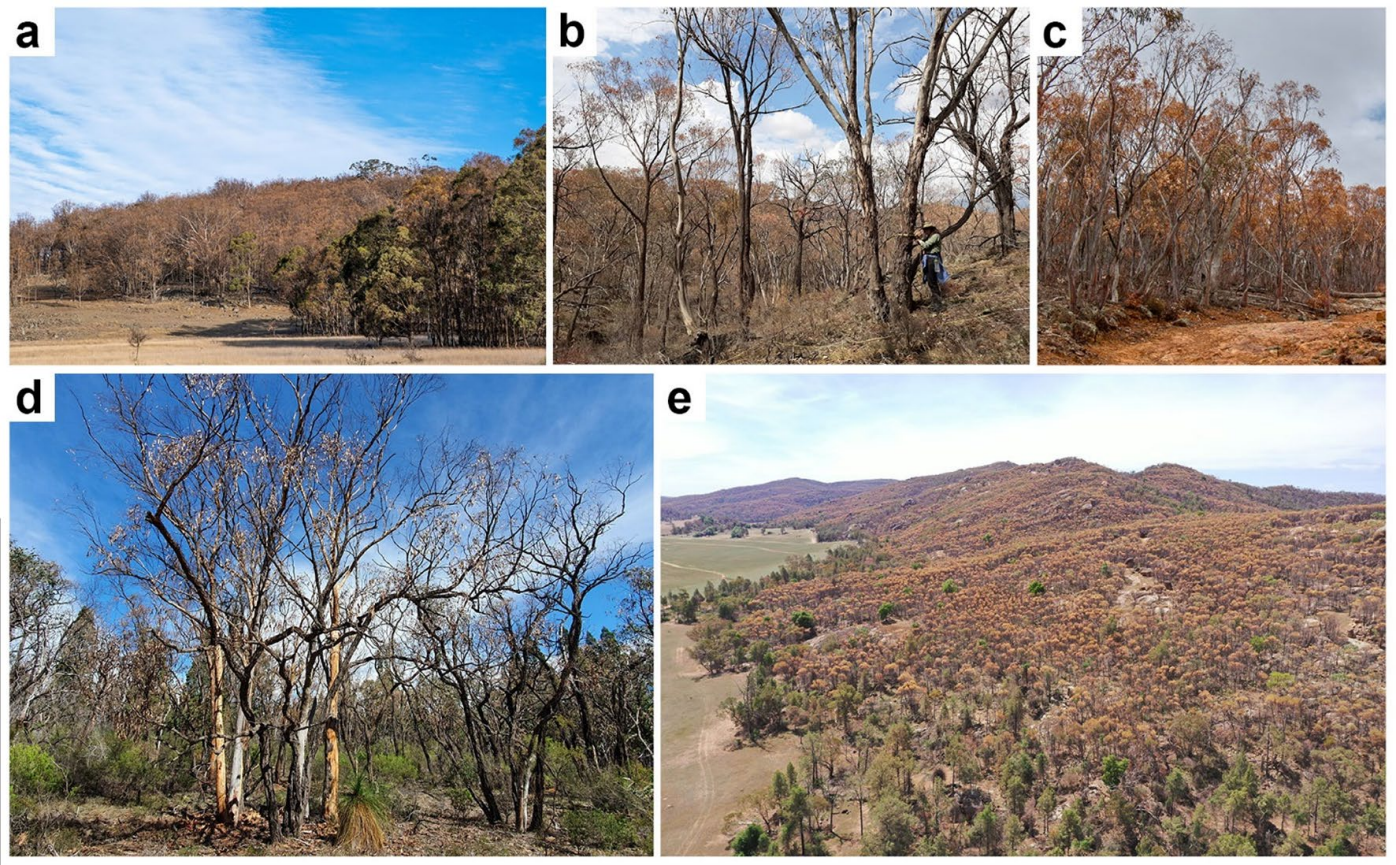
General view of some of the study sites exhibiting extensive canopy dieback: (**a**) Mt Duval, (**b**) Billywillinga, (**c**) Mt Ainslie, (**d**) Munghorn Gap National Park, and (**e**) Eugowra Nature Reserve (see Table 1 for site locations). Photos were taken between November 2019 and February 2020.

**Figure 2:**
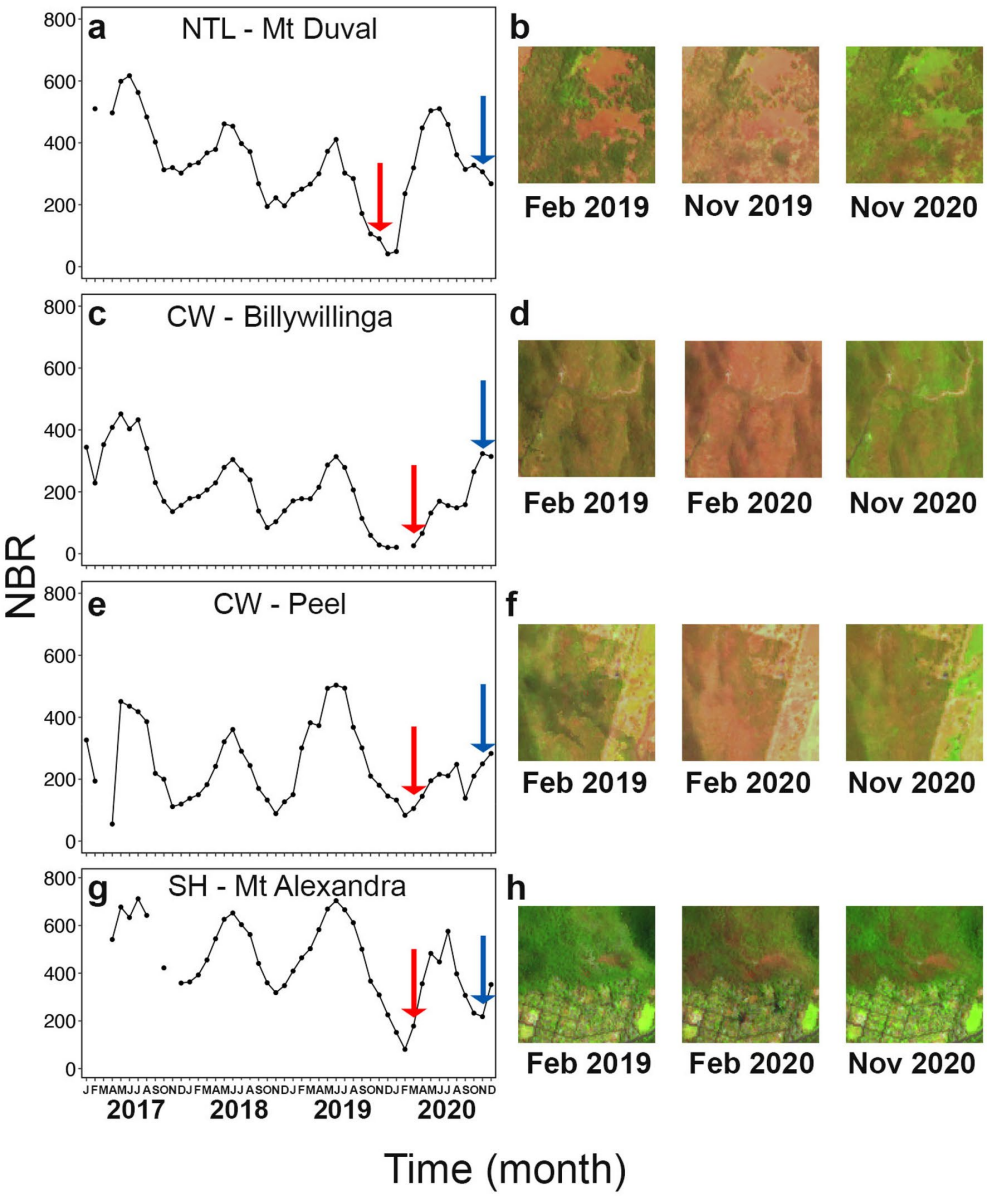
Monthly normalized burn ratio (NBR) from January 2017 to December 2020 measured at four representative sites: (**a**) Mt Duval (in the Northern Table Lands, NTL), (**c**) Billywillinga (in the Central West, CW), (**e**) Peel (in the Central West, CW), and (**g**) Mt Alexandra (in the Southern Highlands, SH). For each site, three satellite images (1 km × 1 km) are representative for the periods before the drought (February 2019), during the drought (November 2019 and February 2020), and post-drought (November 2020). Red and blue arrows indicate first and second sampling campaigns, respectively.

In Australia, 2019 was the driest and hottest year on record (1.52 °C above the average), and was the third consecutive year of drought for many regions across the country^32^. For south-eastern Australia, this extreme drought resulted in massive canopy dieback events^33^, as well as record-breaking wildfires^34,35^. This extreme drought was followed by a period of high precipitation throughout most of 2020, with rainfall above average for much of eastern Australia^32^. In this study, we took advantage of this unprecedented drought event to survey the hydraulic impairment and canopy health of 18 native tree species from varying environments and vegetation types, which were subject to differing drought impacts. Field surveys and measurements were performed at the peak of the drought (late 2019/early 2020; hereafter referred to as “drought”) and after a recovery period characterized by above average precipitation for much of the region (late 2020; hereafter referred to as “post-drought”). Measurements included quantification of native embolism in trees exhibiting different levels of canopy health conditions quantified both on the ground and with satellite imagery. The main objectives of this study were (1) to assess the effect of the drought on tree hydraulics and overall canopy health of several different native tree species with different wood density growing at different sites (see Fig. 1), (2) estimate the recovery (of both hydraulic conductivity and canopy health) after prolonged favorable conditions, and (3) quantify the longer-term impact of the drought by estimating mortality rates both during and after the drought.

## Results

### Satellite imagery

Seasonal variation is evident in the normalized burn ratio (NBR) time series of selected sites, with values typically lower during summer (December–February) and higher during winter (June–August) (Fig. 2). Except for the Peel site (Fig. 2e), there are clear downward trends across 2018 and 2019. The time series also shows that the first field campaign (drought) coincided with extremely low NBR values and there had been no other recent disturbances such as fires (see Fig. 2). The satellite imagery indicates a rapid spectral recovery following increased rainfall at many sites, with NBR values at the time of the second field campaign approaching pre-disturbance values.

### Native loss of conductivity

**Figure 3:**
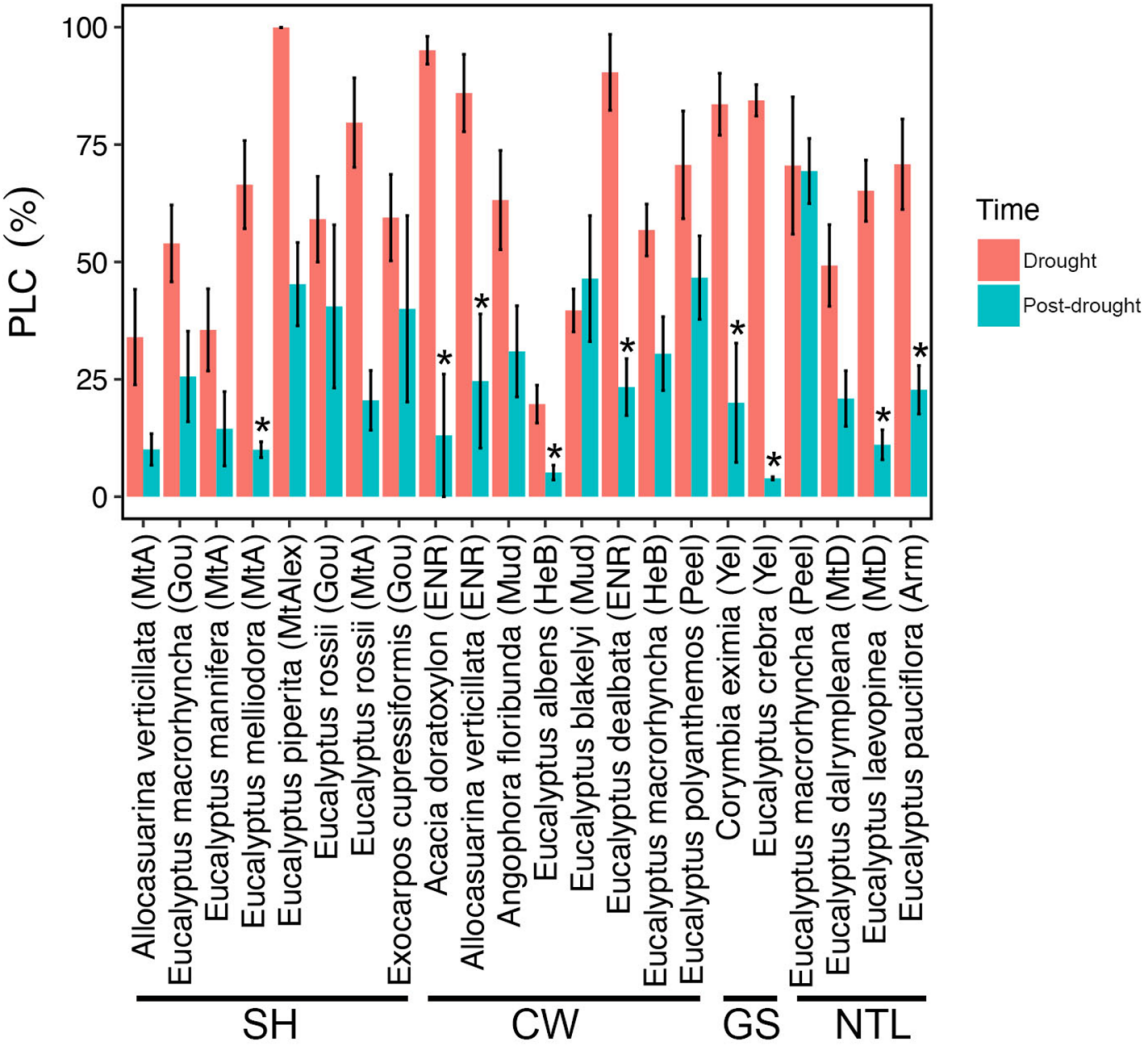
Percent loss of conductivity (PLC, %) during drought (red) and post-drought (light blue) for each species understudy. For each species, the abbreviation in brackets indicates the sampling site (please see Table 1). Species are grouped according to the sampling region: Southern Highlands (SH), Central West (CW), Greater Sydney (GS) and Northern Table Lands (NTL) (see Table 1 for site abbreviations). Values shown are means ± SE (n = 2–10; see also Table S1). Asterisks indicate significant differences (P < 0.05) between PLC measured before and after the recovery.

In the drought phase, percent loss of conductivity (PLC) values varied from 20 to 100%, but only 4 species had PLC values lower than 40% (*A. verticillata* (MtA), *E. albens*, *E. blakelyi* and *E. mannifera*). There was no clear pattern in PLC with MAP across sites: the highest PLC values occurred at both the driest site (ENR: *A. doratoxylon* 95.1 ± 3.0%, *E. dealbata* 90.4 ± 8.1%) and the wettest site (Mt Alexandra: *E. piperita* 99.9 ± 0.0%). We also found no relationship between PLC and the precipitation deficit during drought, calculated as precipitation minus potential evapotranspiration over the two years prior to the measurements (p = 0.12, analyses not shown). There was variation between species at a site as well as among the same species growing at different sites (Fig. 3 and Table S1). For example, *A. verticillata* specimens growing in ENR exhibited significantly higher PLC (86.0 ± 8.2%) than specimens growing at Mt Ainslie (34.0 ± 10.2%). However, *E. macrorhyncha* specimens growing at three different sites showed overall similar PLC (HeB 56.8 ± 5.5%, Peel 70.5 ± 14.6% and Goul 54.0 ± 8.2%). No relationship was found between PLC and mean wood density (p = 0.44, data not shown).

In the post-drought phase, PLC values were overall lower than during the drought phase and showed recovery at most sites (see Fig. 3 and Table S1). Most species recovered to PLC values lower than 40% except for *E. macrorhyncha* (Peel; 69.4 ± 6.9%), *E. polyanthemos* (46.7 ± 8.9%), *E. blakelyi* (46.5 ± 13.4%), *E. piperita* (45.3 ± 8.9%), and *E. rossii* (Goul; 40.5 ± 17.4). However, the same species growing at different sites exhibited different levels of recovery: for example, *E.macrorhyncha* did not recover at Peel, but was able to recover to PLC values of 25–30% at Goul and HeB sites (Fig. 3 and Table S1).

### Canopy health assessment

**Figure 4:**
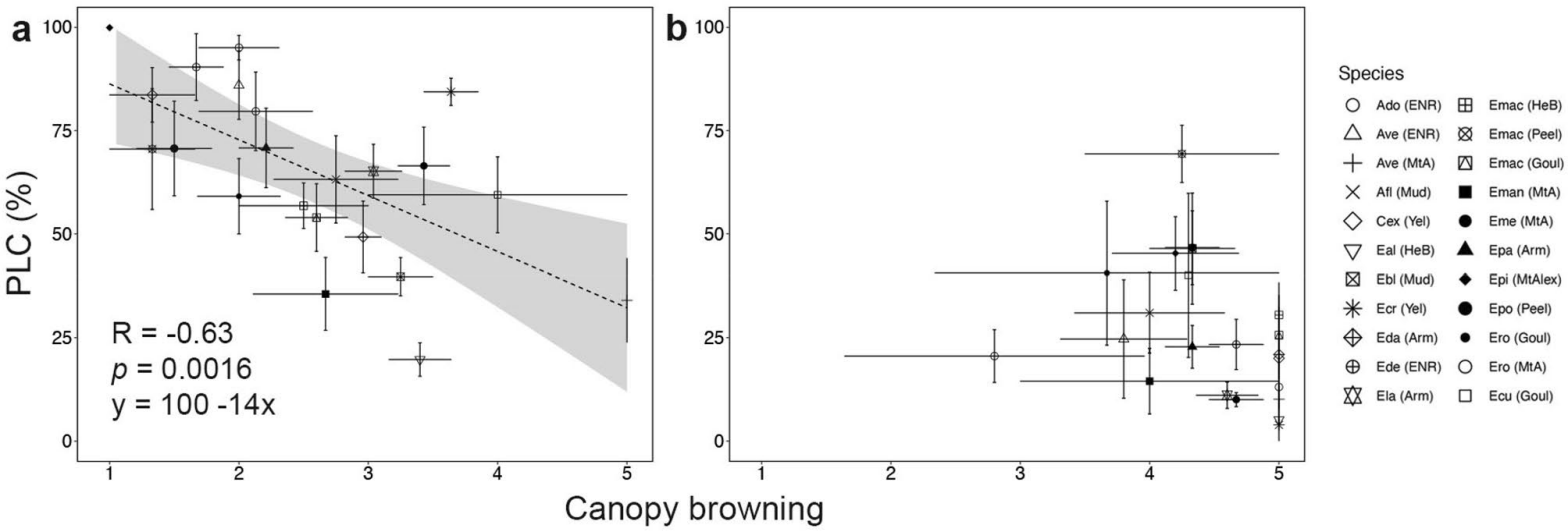
Canopy browning score versus percent loss of conductivity (PLC, %) during drought (**a**) and post-drought (**b**). Each point represents a species at a site; error bars show SE across sampled trees (n = 3–109). The grey shaded area is the confidence interval of the regression line. See references in Table S1 for abbreviations used in the legend.

During the drought measurement period, trees exhibited substantial canopy browning, with the brown canopy largely retained. The canopy browning score (BS) had a significant negative relationship with PLC in this phase, suggesting that canopy BS is a good indicator of loss of hydraulic conductivity (R = − 0.63, P < 0.001; Fig. 4a). During the post-drought phase, BS was higher (3.5–5) than during the drought phase for most species under study, indicating reduced browning (i.e., greener canopies) across the canopy (see Table S1 and Fig. 4). The increase in BS typically resulted from shedding of dead leaves and growth of epicormic resprouts in surviving individuals. During this phase, BS was not significantly correlated with PLC (Fig. 4b).

**Figure 5:**
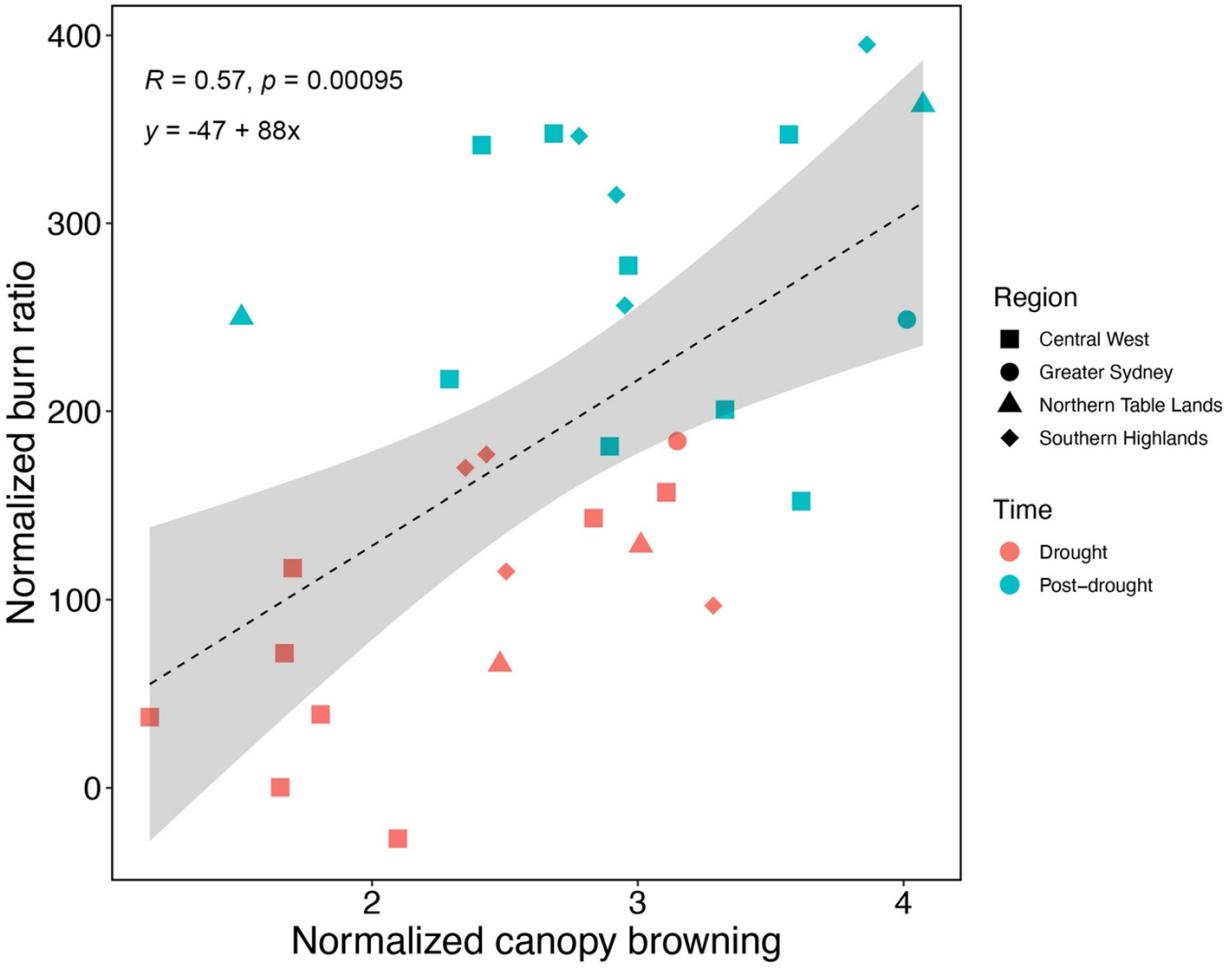
Normalized canopy browning versus normalized burn ratio (NBR) measured at each site during drought (red symbols) and post-drought (light blue symbols). Different symbols indicate the four regions under study: Southern Highlands (diamonds), Central West (squares), Greater Sydney (circles) and Northern Table Lands (triangles). The grey shaded area is the confidence interval of the regression line.

At the site level, normalized canopy browning measured during and after drought was significantly related to NBR values during the month in which each site was visited (R = 0.6, P = 9.5e−0.4; Fig. 5). However, the correlation was stronger during the drought period than during the post-drought phase, suggesting that NBR is a better indicator of canopy browning during periods of severe stress than during recovery.

**Figure 6:**
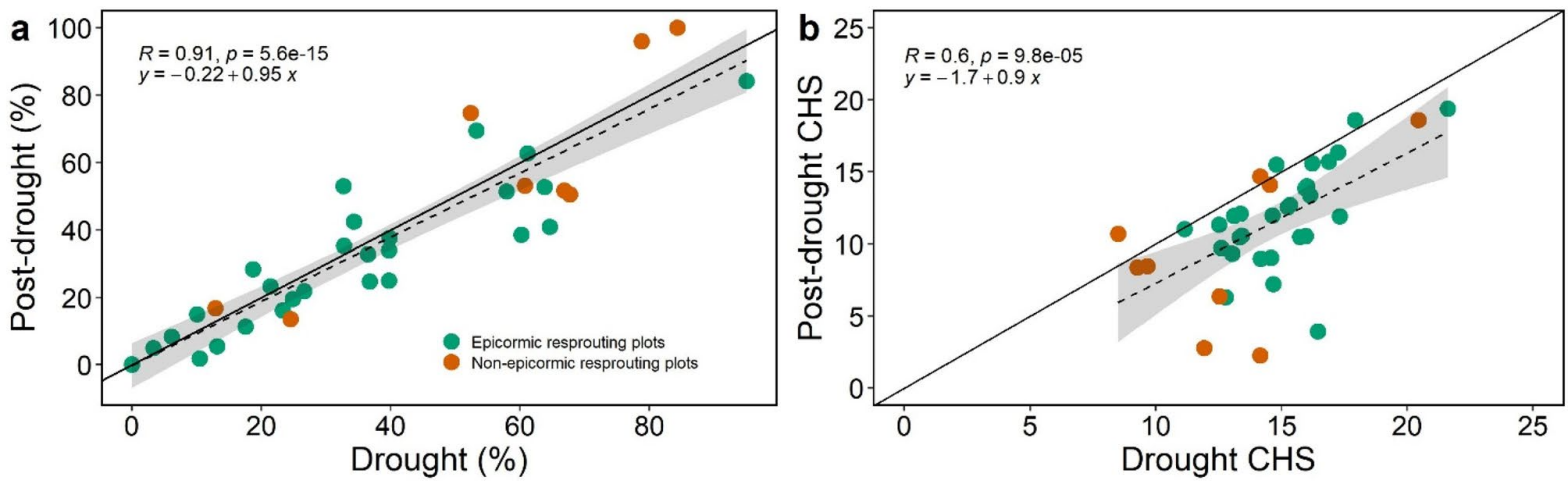
Plots of percentage of tree basal area exhibiting browning scores ≤ 1 (**a**), and weighted basal-area Canopy Health Score (CHS; **b**) measured during drought and post-drought at each plot under study. Colors indicate epicormic (green) and non-epicormic (orange) dominant plots. Dashed line shows linear regression, solid line shows 1:1 line. The grey shaded area is the confidence interval of the regression line.

The increase in BS from drought to post-drought phase is not entirely representative of the change in canopy health because BS is only one component of canopy health score (CHS). Declines in mean CHS occurred due to lower values in other components, including canopy density, crown size, resprouting, and number of dead branches. We observed an increase in spread of CHS scores during post-drought: the frequency of low CHS scores (< 10, poorer canopy conditions) increased in post-drought compared to the drought phase, with most *Eucalyptus* species showing an increase in the frequency of CHS values lower than 5 (Fig. S3). Species showing minimal decline in CHS included the *Callitris* and *Allocasuarina* species and *E. blakelyi*. *Acacia doratoxylon* exhibited the highest increase in the frequency of CHS equal to 0 (no canopy, i.e., from 2.6 to 32.2% between phases; see Fig. S3), indicating a large increase in trees that were dead or dying. The reduction in average CHS over time indicates that, while greenness increased strongly between drought and post-drought campaigns, overall canopy health declined in many cases (Fig. 6b).

CHS increased significantly with DBH during the drought phase in five out of 18 species, suggesting a higher levels of canopy dieback in smaller trees during drought (Fig. S4). In the drought phase, an all-species analysis indicated an overall significant increase in CHS with DBH (Fig. S6a). In contrast, in the post-drought phase, CHS values were independent of DBH in all species under study (Figs. S5 and S6b).

Drought and post-drought percentages of basal area with dead canopy (i.e., BS equal to 0 and 1) showed a one-to-one relationship (P < 0.001; Fig. 6a), indicating species with extensive dieback during the drought phase struggled to re-grow their canopy, even after a prolonged period of above-average rainfall and soil water availability. We found no difference in canopy recovery rates between plots which were dominated by species with the capacity to resprout epicormically after complete defoliation, and those without (Fig. 6a).

## Discussion

This study provides important insights into the impact of an extreme drought event on Australian native tree species growing across a range of forest and woodland environments. The severe 2017–2020 drought, combined with above average temperatures, resulted in extensive crown desiccation and browning for the majority of tree species under study. We observed that canopy browning resulting from the drought was strongly related to branch PLC, providing further evidence that hydraulic failure is related to canopy dieback under natural drought conditions. After a 10–12-month period of higher rainfall, some trees survived and partially recovered while others showed no signs of recovery and were assumed to have died. In trees that survived, leaf area was replaced mainly by epicormic resprouting and PLC was observed to recover by at least 50%. Trees that exhibited complete canopy death during the drought generally did not recover during the subsequent period of favorable rainfall. Canopy browning observed during ground-based observations was also significantly correlated to a satellite derived metric of canopy death, suggesting that NBR will be useful in detecting drought induced canopy death at broader scales.

The severe drought of summer 2019–2020 led to marked hydraulic impairment in most species under study, with PLC values reaching between 19 and 99% (Fig. 3 and Table S1). Reported differences in PLC were both species-specific and site related (e.g., *A. verticillata* growing at different sites exhibited PLC values ranging from 34 to 86%; see Fig. 3 and Table S1). Recent work indicates that diurnal cycles of embolism repair do not occur in eucalypt species and that native PLC measured during the drought phase is indicative of the highest level of water-stress experience by that tree during the drought^31,36^. In the post-drought phase, most trees exhibited lower PLC indicating hydraulic recovery (Fig. 3), although some species still showed critically high values of hydraulic impairment (e.g., PLC: *E. macrorhyncha* 69.4 ± 6.9, *E. blakelyi* 46.5 ± 13.4, *E. cupressiformis* 46.7 ± 8.9; see Fig. 3 and Table S1). Further, the hydraulic recovery observed for some trees may be an overestimation as measurements were made on branches that survived the drought (ignoring the dead ones), and therefore, do not represent the hydraulic status of the whole population. It is assumed that the decline in native PLC between drought and post-drought phases was achieved by growth of new xylem tissue in surviving branches. Recent evidence from in vivo micro-CT observations of *Eucalyptus saligna* support this; plants exposed to a severe drought treatment exhibited high stem PLC with surviving individuals regaining around 70% of the previously lost hydraulic conductivity by growth of new xylem tissue over a six-month period^31^. Hence, we speculate that surviving *Eucalyptus* trees took advantage of the prolonged recovery phase to grow new xylem and recover from drought induced xylem embolism. Again, it is important to note that PLC measurements were collected in surviving trees and therefore recovery in PLC does not translate to impacts on vegetation structure at the plot level, i.e., many trees failed to recover over the 8–12-month period between visits but this mortality in not incorporated in mean PLC for each species.

In this study, we did not observe any relationship between native PLC and site climate variables (e.g., MAP). This is consistent with the convergence of hydraulic safety margins observed across aridity gradients and biomes^37,38^ and the strong relationship between vulnerability to embolism and climate variable observed for eucalypt species^39,40^. Although we do not have vulnerability threshold data for all of the species included in this study, it is likely that species occurring in drier sites are more resistant to embolism than those growing at wetter sites. In this case, a converge in native PLC values would be expected across sites.

In this study, during the drought phase, higher rates of PLC were correlated with higher rates of canopy browning, where trees exhibiting severe hydraulic impairments (PLC > 70%) also showed extensive canopy browning (BS < 2, see Fig. 4a). These results agree with a laboratory-based study reporting a tight correlation between desiccation time and hydraulic safety in 8 *Eucalyptus* species^41^ and with the observations made by Schuldt et al.^21^ after the extreme 2018 summer drought in Central European forests (i.e., high PLC values and widespread leaf discoloration and premature leaf shedding). During the post-drought phase, the regained hydraulic function (PLC < 50%) corresponded to lower rates of canopy browning (BS 3.5–5) and indicated an overall improvement in tree health (Fig. 4b). Reduced canopy browning (BS 3.5–5) also corresponded to higher NBR values for most of our study sites, with a clear improvement from drought to post-drought (Fig. 5). Despite the paucity of direct measurements of hydraulic impairment in eucalypt forests during and after drought events^24^, our results agree with a previous study^40^ reporting native embolism to be positively correlated with canopy dieback even at lower extents of canopy dieback and native embolism (PLC < 26%). The strong relationship between canopy browning and native PLC shown in our findings provides further evidence for the role of hydraulic failure in drought induced mortality and canopy dieback in large trees exposed to an extreme drought.

After a drought event, the growth and general physiological activities of trees are often inhibited rather than the tree suffering immediate death. According to Wu et al*.* (2018), drought legacy responses in deep-rooted forests can manifest up to 4 years after an extreme drought. This might leave trees highly vulnerable to secondary drought events and/or insect and fungal pathogen attacks^42^. Although we were limited in our ability to fully explore the legacy effects of drought on the trees under study, in the post-drought phase (8–10 months after the drought event ended), the frequency distribution of CHS tended to flatten out, and we observed an overall decline in CHS for the majority of species. Accordingly, species such as *A. floribunda*, *E. crebra* and *E. melliodora* exhibited relatively strong canopy health recovery (Fig. S3) with an increase in CHS higher than 15, whereas *A. doratoxylon* exhibited the highest increase in the frequency of CHS equal to 0 (i.e., from 2.6 to 32.2% between phases; see Fig. S3), indicating an increase in presumably dead trees. The decline in CHS (Fig. 6b) occurs despite the strong recovery in greenness seen in both ground observations and satellite imagery. It appears that while trees were able to increase the proportion of green leaves in the post-drought period, often via epicormic resprouting, the overall canopy health declined despite favorable rainfall over an 8-month period. This is consistent with studies showing a legacy response to drought, in which plant health indices continue to decline even after release from drought^40,43^.

While defining whether a tree is dead or not in species with strong resprouting capacity remains difficult, we can assume that trees displaying extensive canopy dieback (BS ≤ 1) after an eight-month period of favorable rainfall are either already dead, or at very high risk of death. Moreover, the one-to-one relationship between drought and post-drought percentages of basal area with BS ≤ 1 suggests that trees with a severely compromised canopy immediately after a drought event did not manage to recover even with prolonged favorable water availability (Fig. 6a). Across sites, the percentages of tree basal area exhibiting dead canopies were notably higher (between 35 and 100%; Fig. 6a) than previously reported by other studies on *Eucalyptus* dominated forests under non-drought conditions (from 0 to 27.5%)^44–46^.

Some studies have found that larger trees are more vulnerable to drought than smaller ones^12,26^, while others have predicted that younger and presumably smaller trees have higher drought-induced mortality in secondary forest landscapes^47^. However, we did not observe a relationship between DBH and CHS for the majority of species growing in the forests under study (Figs. S4 and S5). In five *Eucalyptus* species, we observed a positive relationship between DBH and CHS immediately following drought (Fig. S4), suggesting that smaller trees in these species were at greater risk of canopy dieback than larger trees, but this relationship typically did not persist in the post-drought period (Fig. S5), indicating that the small trees experienced similar longer-term impacts to the larger trees.

In our study, most plots were dominated by trees with the ability to epicormically resprout (i.e., regenerate after severe loss of biomass by sprouting from meristematic tissue). The role of resprouting species in mediating the ecosystem response to drought stress is still rarely considered^48^. Whether ecosystems dominated by resprouting species are more resilient to drought-stress, or can recover more quickly, than ecosystems dominated by non-resprouting species is still controversial and contrasting results have been reported in previous studies^49,50^. Our results indicate that plots dominated by epicormic resprouters suffered overall lower, although still significant, percentages of basal area tree loss than plots dominated by non-epicormic resprouters (Fig. 6a). Hence, under such extreme drought events, species with differing response to defoliation (see also Material and methods) seems to be differentially impacted by canopy dieback.

To date, this is one of few studies reporting direct hydraulic measurements after natural drought, and one of the even fewer studies to track physiological and structural recovery following release from drought. These unique observations allowed us to investigate the limits of tree hydraulic function to drought-induced mortality under different field conditions over an extended geographical area, and to test tree survival after a return to favorable conditions. Our results provide further evidence that hydraulic failure is a principal causal mechanisms of tree mortality during extreme drought^7,9^, and are consistent with previous field-based observations^1,19–21,24^ reporting high rates of hydraulic impairment (PLC) during drought. Our results also capture the legacy effects of drought, with some lower levels of canopy health persisting 8–10 months after trees were released from drought stress. A considerable proportion of trees showed no signs of recovery at the majority of sites, suggesting a high level of tree mortality resulting from the drought. Recovery of the hydraulic system occurred slowly in surviving individuals, most likely occurring through growth of new xylem tissue in surviving individuals. The 2019 drought is exactly the type of event that is predicted to become more frequent in the future across Australia, and our data on resprouting mechanisms carry clear implications for conservation, restoration, forestry, and land management in these forest ecosystems. Further monitoring of forest and woodland environments in eastern Australia will be required to evaluate the resilience of these systems to extreme drought events. This includes a more systematic approach to monitoring forest health that will allow for improved validation of satellite projects and real-time data necessary to ecological forecasting efforts.

## Material and methods

### Study sites and species

All measurements were performed at 15 native forest sites in eastern Australia (across New South Wales and the Australian Capital Territory; see Table 1). The study sites were identified through a citizen science effort, the Dead Tree Detective^33^, which allowed us to locate forest sites across the region experiencing significant canopy dieback (see Figs. 1 and 2). Sampling sites fall within four distinct geographical areas: Northern Table Lands (NTL), Greater Sydney (GS), Central West (CW) and Southern Highlands (SH) (see Table 1). From April 2019 to January 2020, all sites experienced a prolonged dry period exacerbated by extremely high temperatures between December 2019 and January–February 2020 (up to 40–43 °C) (see Fig. S1). Sites had not been recently impacted by fires and did not burn in the 2019–2020 Black Summer bushfires^34,35^. In both New South Wales and the Australian Capital Territory, summer 2019–2020 had the lowest rainfall (55% below average; Fig. S1) and highest temperatures on record (mean temperature 1.52 °C above average). From February–March 2020, above-average precipitation was observed across the region (14% above average; see Table 1 and Fig. S1). At the sampling sites, MAP ranges between 580 and 910 mm. Across all sites, the mean reduction in annual rainfall over the two years prior to measurements ranged from 22 to 50%, while annual rainfall in 2020 was 100–135% of MAP (Fig. S1).

**Table 1:** General information of sampling sites (with abbreviations used throughout the manuscript), species measured at each site and their characteristics (exposure, GPS coordinates, elevation, mean annual rainfall, vegetation type and basal area).

Geographic area	Site	Site abbreviation	GPS coordinates	Elevation (m)	Mean annual rainfall (mm)	Exposure	Vegetation type^1^	Species	Measurements^2^	BA^3^ (%)
Northern Table Lands	Armidale, NSW	Arm	30° 29ʹ S, 151° 38ʹ E	983	716	S	GW	*Eucalyptus pauciflora* Sieber ex Spreng	PLC, CHS	95.1
	Mt Duval, NSW	MtD	30° 25ʹ S, 151° 37ʹ E	1124	776	S	GW	*Eucalyptus dalrympleana* Maiden	PLC, CHS	69.4
				1160		S	GW	*Eucalyptus laevopinea* R.T.Baker	PLC, CHS	27.6
Greater Sydney	Yellomundee, NSW	Yel	33° 39ʹ S, 150° 39ʹ E	50	611	NE	DSF	*Corymbia eximia* (Schauer) K.D.Hill & L.A.S.Johnson	PLC, CHS	32.0
						NE	DSF	*Eucalyptus crebra* F. Muell	PLC, CHS	22.6
						NE	DSF	*Exocarpos cupressiformis* Labill	CHS	24.2
Central West	Eugowra Nature Reserve, NSW	ENR	33° 17ʹ S, 148° 20ʹ E	410	568	W	DSF	*Acacia doratoxylon* A.Cunn	PLC, CHS	10.6
						W	DSF	*Allocasuarina verticillata* (Lam.) L. A. S. Johnson	PLC, CHS	4.8
						W	DSF	*Callitris endlicheri* (Parl.) F.Muell	CHS	40.6
						W	DSF	*Eucalyptus dealbata* A. Cunn. ex Schauer	PLC, CHS	38.4
	Peel, NSW	Peel	33° 19ʹ S, 149° 38ʹ E	710	654	E	DSW	*Eucalyptus macrorhyncha* F. Muell. ex Benth	PLC, CHS	55.3
						E	DSW	*Eucalyptus melliodora* A. Cunn. ex Schauer	CHS	5.9
						E	DSW	*Eucalyptus polyanthemos* Schauer	PLC, CHS	20.0
	Nangar National Park, NSW	NNP	33° 24ʹ S, 148° 29ʹ E	450	676	NW	DSF	*Acacia doratoxylon* A.Cunn	CHS	7.7
						NW	DSF	*Callitris endlicheri* (Parl.) F.Muell	CHS	9.1
						W	DSF	*Eucalyptus albens* Benth	CHS	4.7
						W	DSF	*Eucalyptus dealbata* A. Cunn. ex Schauer	CHS	25.6
						W	DSF	*Eucalyptus sideroxylon* A.Cunn. ex Woolls	CHS	46.7
	Mud Hut Road, NSW	Mud	32° 25ʹ S, 149° 40ʹ E	540	654	flat	GW	*Angophora floribunda* (Sm.) Sweet	PLC, CHS	14.7
						flat	GW	*Eucalyptus blakelyi* Maiden	PLC, CHS	68.7
						flat	GW	*Eucalyptus melliodora* A. Cunn. ex Schauer	CHS	0.6
	Henry Bayly, NSW	HeB	32° 36ʹ S, 149° 34ʹ E	610	712	W	DSW	*Angophora floribunda* (Sm.) Sweet	CHS	6.4
						W	DSW	*Eucalyptus albens* Benth	PLC, CHS	33.5
						W	DSW	*Eucalyptus blakelyi* Maiden	CHS	5.0
						W	DSW	*Eucalyptus macrorhyncha* F. Muell. ex Benth	PLC, CHS	47.0
	Munghorn Gap National Park, NSW	MGNP	32° 24ʹ S, 149° 49ʹ E	690	690	W	DSF	*Callitris glaucophylla* Joy Thomps. & L.A.S.Johnson	CHS	12.1
						W	DSF	*Eucalyptus albens* Benth	CHS	0.7
						W	DSF	*Eucalyptus blakelyi* Maiden	CHS	15.9
						W	DSF	*Eucalyptus macrorhyncha* F. Muell. ex Benth	CHS	20.6
						W	DSF	*Eucalyptus rossii* R. T. Baker & H. G. Sm	CHS	48.4
	Billywillinga, NSW	Billy	33° 17ʹ S, 149° 26ʹ E	800	643	W	GW	*Eucalyptus albens* Benth	CHS	1.7
						W	GW	*Eucalyptus blakelyi* Maiden	CHS	18.5
						W	GW	*Eucalyptus goniocalyx* F.Muell. ex Miq	CHS	55.1
						W	GW	*Eucalyptus macrorhyncha* F. Muell. ex Benth	CHS	16.8
						W	GW	*Eucalyptus melliodora* A. Cunn. ex Schauer	CHS	0.2
						W	GW	*Eucalyptus polyanthemos* Schauer	CHS	7.5
	Wattle Flat, NSW	WaF	33° 8ʹ S, 149° 41ʹ E	950	637	NW	DSF	*Eucalyptus albens* Benth	CHS	0.9
						NW	DSF	*Eucalyptus goniocalyx* F.Muell. ex Miq	CHS	68.0
						NW	DSF	*Eucalyptus macrorhyncha* F. Muell. ex Benth	CHS	29.3
						NW	DSF	*Eucalyptus melliodora* A. Cunn. ex Schauer	CHS	2.9
						NW	DSF	*Eucalyptus polyanthemos* Schauer	CHS	2.7
Southern Highlands	Goulburn, NSW	Goul	34° 45ʹ S, 149° 45ʹ E	730	632	SW-W	DSF	*Eucalyptus macrorhyncha* F. Muell. ex Benth	PLC, CHS	13.7
						SW-W	DSF	*Eucalyptus mannifera* Mudie	CHS	29.4
						SW-W	DSF	*Eucalyptus rossii* R. T. Baker & H. G. Sm	PLC, CHS	53.5
						SW-W	DSF	*Exocarpos cupressiformis* Labill	PLC, CHS	0.5
	Mt Ainslie, ACT	MtA	35° 12ʹ S, 149° 9ʹ E	700	630	S	GW	*Allocasuarina verticillata* (Lam.) L. A. S. Johnson	PLC, CHS	2.1
						S	GW	*Eucalyptus bridgesiana* R.T.Baker	CHS	23.3
						S	GW	*Eucalyptus macrorhyncha* F. Muell. ex Benth	CHS	26.1
						S	GW	*Eucalyptus mannifera* Mudie	PLC, CHS	43.1
						S	GW	*Eucalyptus melliodora* A. Cunn. ex Schauer	PLC, CHS	2.5
						S	GW	*Eucalyptus rossii* R. T. Baker & H. G. Sm	PLC, CHS	1.0
	Mt Majura, ACT	MtM	35° 13ʹ S, 149° 10ʹ E	650	611	Flat	GW	*Eucalyptus blakelyi* Maiden	CHS	14.1
						Flat	GW	*Eucalyptus bridgesiana* R.T.Baker	CHS	3.9
						Flat	GW	*Eucalyptus melliodora* A. Cunn. ex Schauer	CHS	57.2
						Flat	GW	*Eucalyptus pauciflora* Sieber ex Spreng	CHS	20.3
						Flat	GW	*Exocarpos cupressiformis* Labill	CHS	3.8
	Mt Alexandra, NSW	MtAlex	34° 26ʹ S, 150° 27ʹ E	700	933	S-SE	DSF	*Eucalyptus piperita* Sm	PLC, CHS	50.9
						S-SE	DSF	*Eucalyptus sieberi* L.A.S.Johnson	CHS	30.3

A list of measurements performed for each species/site is also given.

^1^GW, DSW and DSF corresponds to the vegetations types Grassy Woodlands, Dry Sclerophyll Woodlands, and Dry Sclerophyll Forests, respectively.

^2^PLC and CHS are percentage loss of conductivity and canopy health score, respectively.

^3^BA is the contribution in basal area (%) of each species at each site.

The study consisted of two sets of measurement campaigns, which were performed (1) during the extreme drought of summer 2019–2020 (i.e., February–March 2020) and (2) after a period of recovery, characterized by above average precipitation (i.e., November–December 2020). All measurements aimed to estimate the effect of drought and included native percent loss of hydraulic conductivity (PLC) and an assessment of canopy dieback, as well as the overall canopy health (see Table 1).

Each site was characterized by different exposures and elevation (see Table 1) and supported different tree species. At each site, two to four circular plots (ca. 30 m diameter) were established, and canopy health scores were estimated for the dominant tree species (1–5 species per site, 78.8–99.7% of basal area) (Table 1). In total, 24 species were included in the observations (Table 1). Of these 24 species, most were eucalypts, which incorporates the *Eucalyptus*, *Angophora* and *Corymbia* genera. Additional species included *Acacia doratoxylon*, *Allocasuarina verticillata, Exocarpos cupressiformis* (a root hemiparasite) and two evergreen conifers, *Callitris endlicheri* and *C. glaucophylla*. ﻿Species identification was carried by Anthea Challis, and permission for sampling was granted by the local authorities as well as private landholders. The majority of our study species are able to recover following complete defoliation from disturbances, such as drought and fire, by resprouting new foliage on the bole and branches of the tree via epicormic resprouting^51^. While many species are capable of resprouting to some extent following some degree of defoliation^52^, *A. doratoxylon* does not typically resprout at all following complete defoliation, and *A. verticillata, E. cupressiformis* and one of the eucalypt species, *Eucalyptus pauciflora*, only recover via basal resprouting.

Drought PLC and canopy health data collected for the species *Eucalyptus dalrympleana*, *E. laevopinea* and *E. pauciflora* are part of a previous study published by Nolan et al. (2021) (measurements were done in November 2019).

No voucher specimens of the plant material under study were collected and deposited in a publicly available herbarium. All measurements were carried out in accordance with institutional, national, and international guidelines and legislation.

### Canopy health assessment

On the plots established at each site, tree diameter at breast height (DBH) was measured on each tree belonging to the group of dominant species and tree canopy health condition (Canopy Health Score; CHS) was assessed for each tree with DBH > 5 cm. DBH for trees with multiple stems was calculated as $\surd {\Sigma }_{i=1}^{n}{d}_{i}^{2}$ (d = stem diameter (cm), according to the Australian National Carbon Accounting System^53^). In total, 1300 trees were collectively measured across the study sites (18–270 trees per species). To assess the CHS, we used a score based on Stone et al*.* (2008) and previously used by Nolan et al. (2021), which involves scoring trees from 0 to 5 for a series of crown attributes: crown size and shape, crown foliar density, dead branches, tree epicormic growth and leaf discoloration/browning. CHS values were assessed by the same two observers across all sites to ensure consistency. A final crown health score was obtained by summing each of the constituent crown attribute scores to obtain values from 0 (i.e., a dead tree with no leaves remaining) to 25 (i.e., a healthy tree) (see Fig. S2 for a practical reference and Table S2 for the definition of each component).

For each plot and species, we calculated the percentage of basal area (BA) with dead canopy (i.e., with a browning score of 0 or 1) during both drought and post-drought as an estimated metric of mortality. For the browning score, a value of 0 represents a tree with no canopy retained, while a value of 1 represents a tree where some canopy is retained, but it has completely died (i.e., the canopy is brown in color). We also calculated a normalized plot-level browning score, as ${\Sigma }_{i=1}^{n}({BA}_{i}* {BS}_{i})/{\Sigma }_{i=1}^{n}{BA}_{i}$, and a normalized plot-level Canopy Health Score, as ${\Sigma }_{i=1}^{n}({BA}_{i}* {CHS}_{i})/{\Sigma }_{i=1}^{n}{BA}_{i}$, where for both equations *i* represents individual trees within the plot. Plots were also divided into two groups: epicormic resprouting plots and non-epicormic resprouting plots (i.e., when at least 20% of basal area is not resprouting).

### Satellite imagery

We compared the ground-based site browning score with satellite estimates of browning. Google Earth Engine^55^ was used to construct monthly median composite images from Sentinel-2 level 1C data from 2017 to 2020. 1 km by 1 km image ‘chips’ of plots and the surrounding area were created for February 2019 (as a pre-drought reference) and the months corresponding to the field visits. A normalized burn ratio (NBR) was calculated (NBR = (band 8 – band 12)/(band 8 + band 12)) and the average NBR values were extracted for each plot (Fig. 2). Plot values were computed by selecting all 10 m pixel centroids that intersected with the circular plot and calculating the mean. Although NBR was initially developed to highlight burnt areas, it is commonly used in forest disturbance studies more generally, due to its greater sensitivity to forest structure^56^.

### Native loss of hydraulic conductivity

For 18 species (see Table S1), percent loss of xylem hydraulic conductivity (PLC) measurements were performed on trees located near the CHS plots during both drought and post-drought visits. Where possible, the same trees were measured for drought and post-drought time points, although in some instances this was not possible because trees had shown no signs of recovery. In these cases, surviving trees were substituted for trees that had not recovered. Unfortunately, PLC measurements could not be performed for all 24 species included in plot surveys due to either sampling or methodical constraints. For each species, three large branches (> 1.5 m long) per tree (*n* reported in Table S1) were collected, wrapped in large black plastic bags containing wet paper towels to stop transpiration and transported to the laboratory. We collected branches that were present during the drought (i.e., 2–3 years old) and avoided newly grown and/or resprouted stems. In the laboratory, samples were placed with their cut ends in water and recut to allow relaxation of internal tensions^57^. For each branch, 4–5 terminal branches were cut under water, and trimmed several times with a sharp razorblade to gradually release tension^57^ and obtain 8–15 cm long segments.

Samples were then connected to a digital liquid flow meter (Liqui-Flow L10, Bronkhorst High-Tech BV, Ruurlo, Gelderland, The Netherlands) and perfused with distilled and degassed water containing 2 mmol KCl and 1 mmol CaCl and filtered at 0.2 μm. The initial hydraulic conductivity (K_i_) was measured at 0.002 MPa, and flushing was done for 15 min at 0.2 MPa to remove embolism. After flushing, the flow rate was measured again at 0.002 MPa (final hydraulic conductivity, K_f_). Flushing was repeated until measurements showed no further increase in flow rate. All measurements were conducted at room temperature (ca. 21 °C), and percent loss of conductivity (PLC) was calculated as (1 – K_i_/K_f_) * 100.

For each tree used for PLC measurements, the canopy dieback was estimated by assessing the extent of canopy browning (i.e., browning score, BS) by giving a score from 1 to 5, where 1 and 5 indicate a completely brown and a completely healthy canopy with no visible discoloration, respectively. A score of 0 was given to trees with no canopy. This score was based on Stone et al. (2008) and previously used by Nolan et al. (2021) (see Fig. S2 for a practical reference). BS were assessed as this best reflects the short-term response to the drought, which is most likely to align with PLC measurements.

Meteorological data were obtained from the Australian Water Availability Project (AWAP) dataset^58^. Gridded data were extracted from the AWAP dataset based on the co-ordinates of each site. Mean annual precipitation (MAP) was calculated based on data over the period 1971–2020. Rainfall deficit was calculated as actual rainfall minus potential evapotranspiration, with the latter calculated using the Thornthwaite equation based on temperature and latitude. We tested for correlations of PLC with MAP, mean rainfall deficit and the actual rainfall deficit during the drought period 2018–2019. PLC values were also correlated with mean wood density values for each species obtained from the Austraits database^59^.

### Statistics

Correlation analysis was carried out using the Pearson product-moment correlation (PLC *versus* BS). Differences between PLC and canopy BS during drought and post-drought periods for each species located within a region were tested using a Student t-test. To assess plot-level recovery, we calculated the percentage of basal area with a dead canopy (i.e., with a BS of 0 or 1) during both drought and post-drought phases and regressed the two scores against each other. To test for relationships between Canopy Health Scores and tree DBH, the following nonlinear model was fitted for each species to capture the asymptotic nature of the data: Canopy Health Score = a/(1 + b/DBH)^24^. The model parameters a and b were fit using nonlinear least squares regression. Data were checked by visual examination of residual errors to ensure that data were normally distributed and that there was no relationship between residuals and predictors. All statistical analyses were undertaken in R 3.5.0 (R Development Core Team, 2018).

## Supplementary Information

Supplementary Information.

## Data Availability

The datasets used during the current study are available from the corresponding author on reasonable request.
