# Supplementary material for: Canopy dieback and recovery in Australian native forests following extreme drought
Source: Sci Rep. 2022 Dec 14;12:21608. doi: 10.1038/s41598-022-24833-y (PMC9751299; doi:10.1038/s41598-022-24833-y)
Supplement: Supplementary file 1 — Supplementary Information. [file 41598_2022_24833_MOESM1_ESM.docx]

Supplementary Material


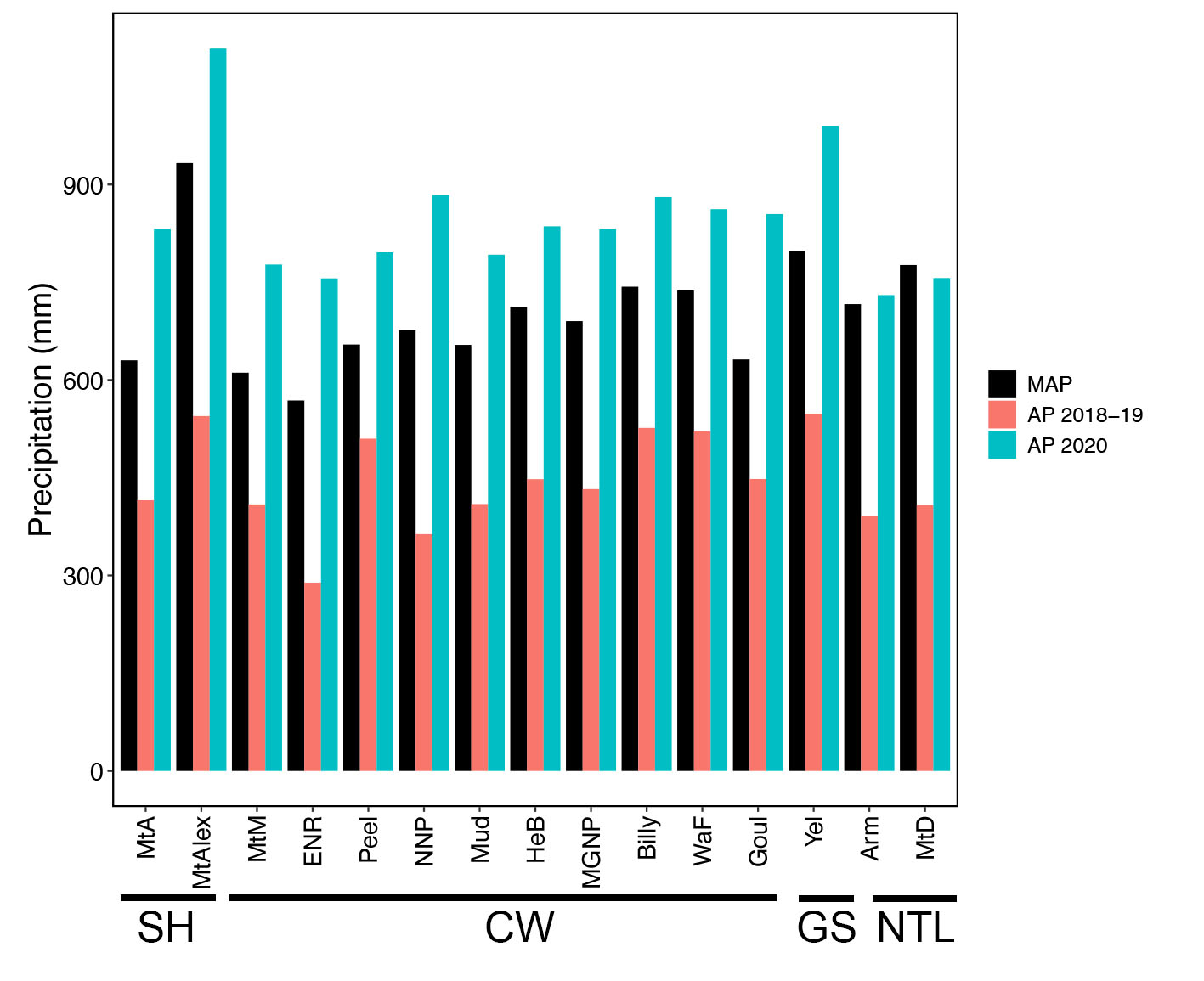


**Figure S1.** Mean annual precipitation (MAP; averaged over 1971 to 2019; black bars), annual precipitation averaged over 2018 and 2019 (AP 2018-19; red bars) and annual precipitation averaged in 2020 (AP 2020; light blue bars) of each site under study. Sites are grouped according to the sampling region: Southern Highlands (SH), Central West (CW), Greater Sydney (GS) and Northern Table Lands (NTL) (see Table 1 for site abbreviations).

**
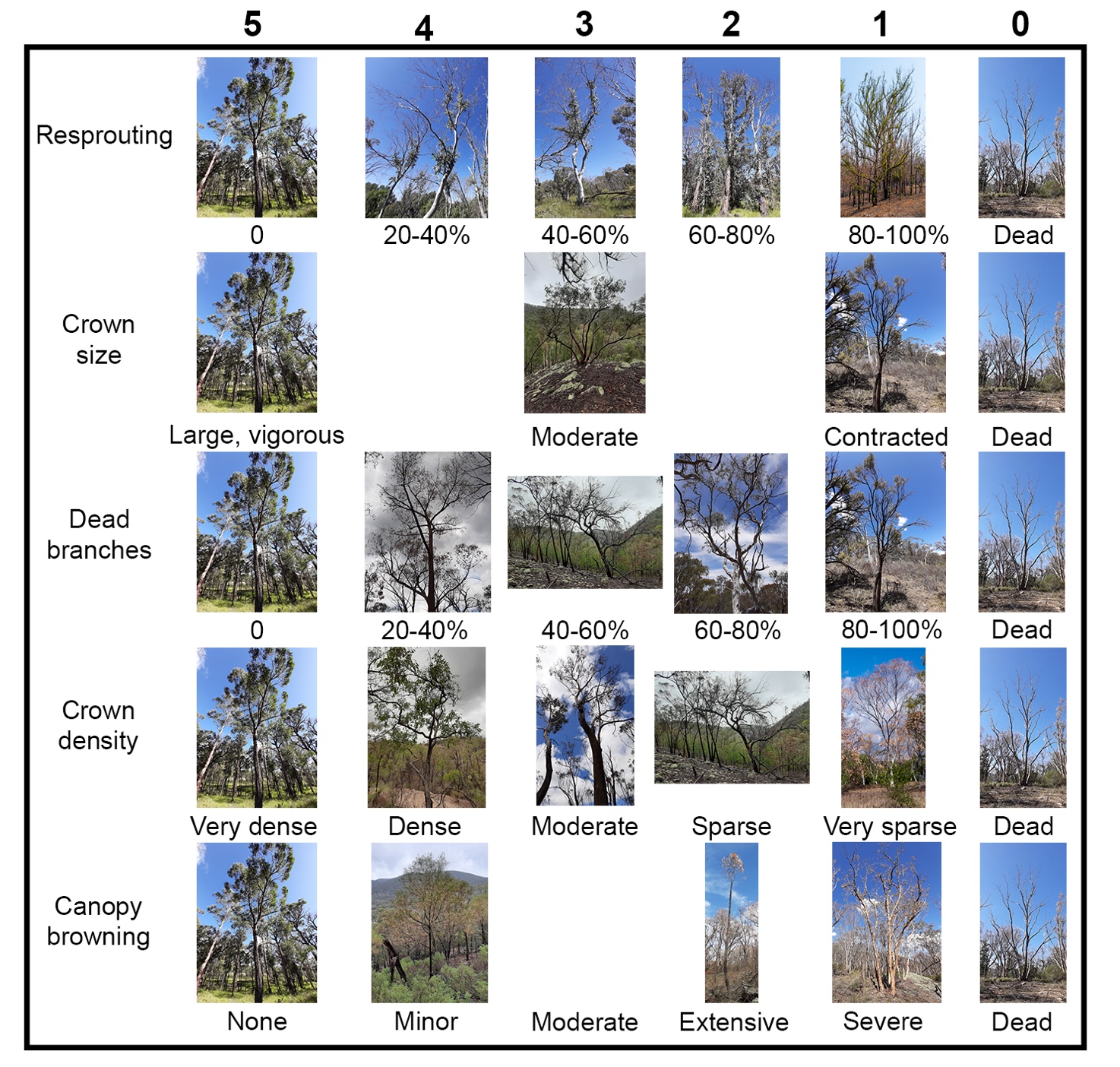
**

**Figure S2.** Practical representation of how the Canopy Health Score was applied, which consists in scoring trees from 1 to 5 for a series of crown attributes: crown epicormic growth (resprouting), crown size and shape, dead branches, crown foliar density, and leaf discoloration/browning. A value equal to 0 was given to dead trees. A final crown health score was obtained by summing each of the constituent crown attribute scores to obtain values from 0 (dead tree) to 25 (healthy tree).

**
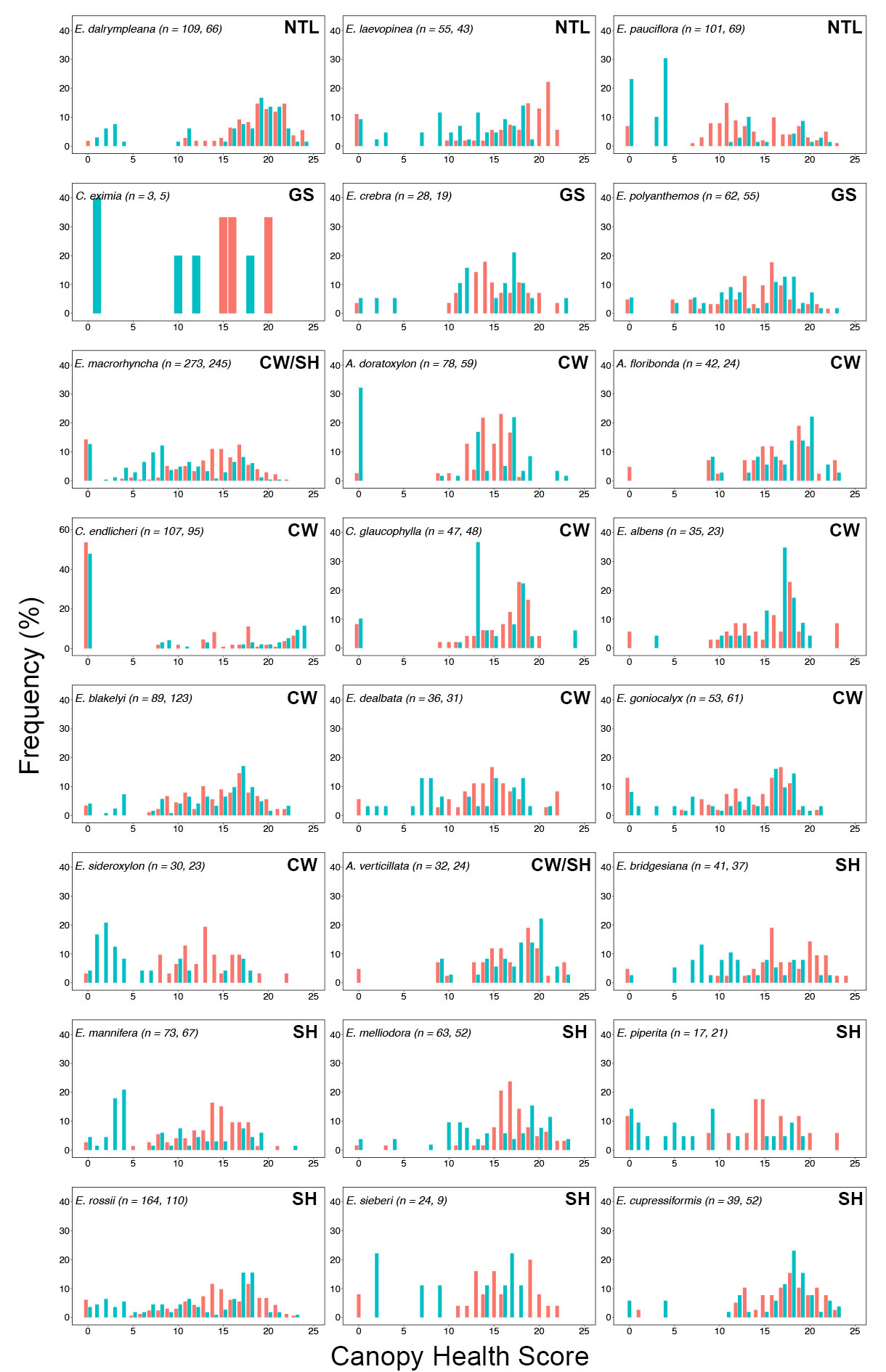
**

**Figure S3.** Frequency of CHS (%) measured during drought (red bars) and post-drought (light blue bars) of each species under study. Species are ordered according to the sampling region: Northern Table Lands (NTL), Greater Sydney (GS), Central West (CW) and Southern Highlands (SH). In brackets, the number of measured trees (n) for drought and post-drought, respectively.


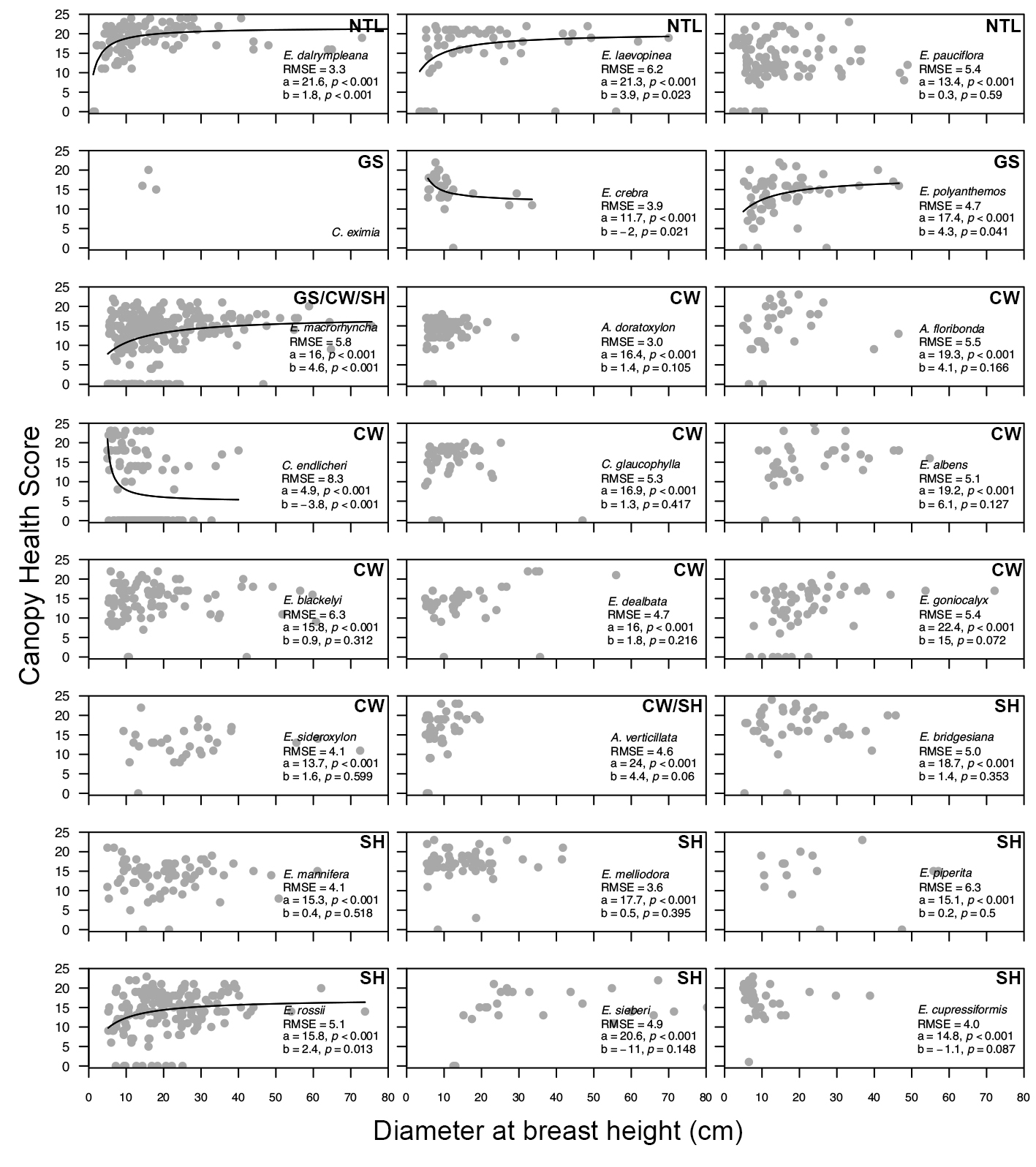


**Figure S4.** Canopy Health Score (CHS) *versus* tree diameter at breast height (DBH, cm) measured during drought for all species under study. Each point corresponds to a single specimen. Higher CHS indicates healthier trees, while scores of 0 indicate completely defoliated trees (please see also Figure S2). RMSE is the root mean square error of the fitted regression model. Species are ordered according to the sampling region: Southern Highlands (SH), Central West (CW), Greater Sydney (GS) and Northern Table Lands (NTL).

**
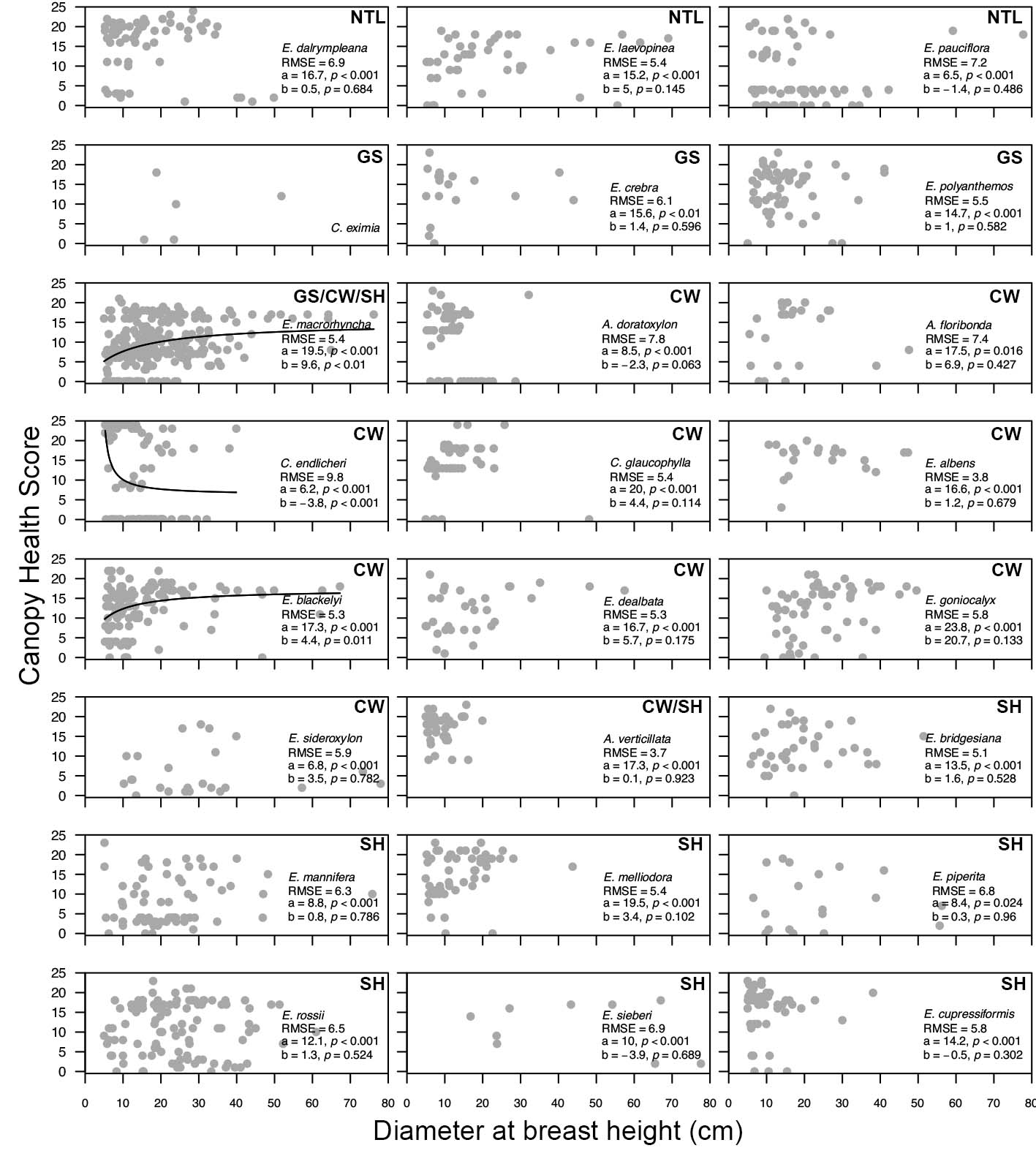
**

**Figure S5.** Canopy Health Score (CHS) *versus* tree diameter at breast height (DBH, cm) measured during the post-drought phase for all species under study. Each point corresponds to a single specimen. Higher CHS indicates healthier trees, while scores of 0 indicate completely defoliated trees (please see also Figure S2). RMSE is the root mean square error of the fitted regression model. Species are ordered according to the sampling region: Southern Highlands (SH), Central West (CW), Greater Sydney (GS) and Northern Table Lands (NTL).

**
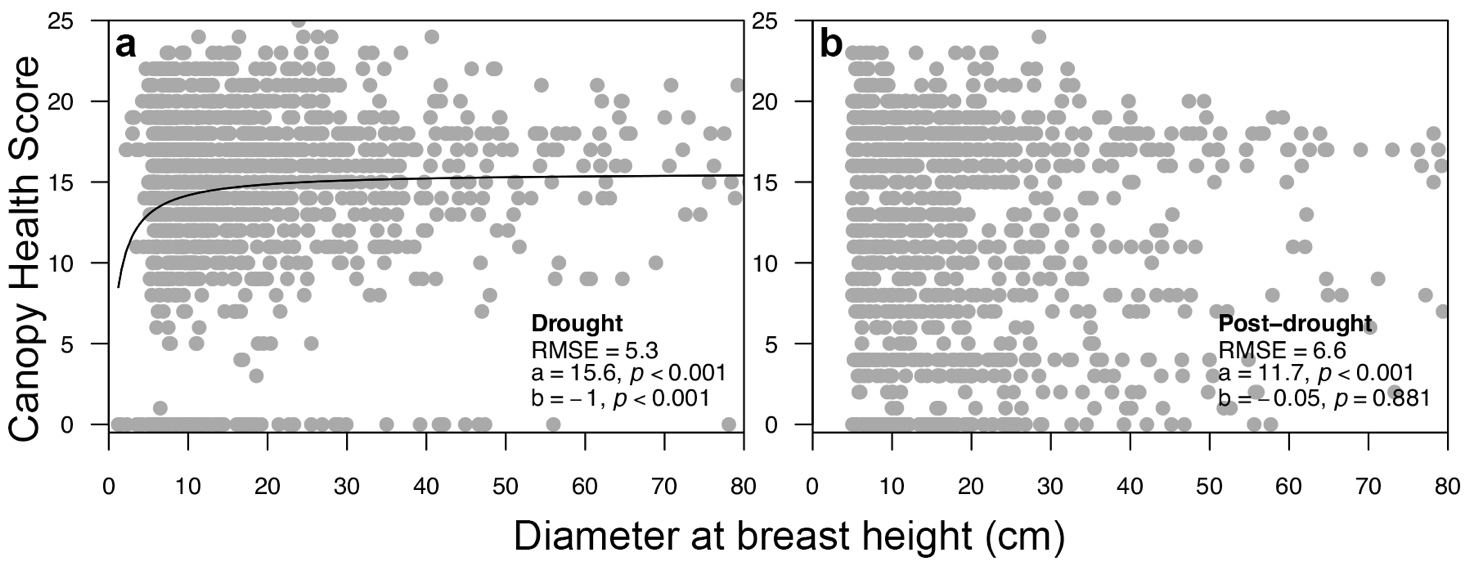
**

**Figure S6.** Canopy Health Score (CHS) *versus* tree diameter at breast height (DBH, cm) measured during drought **(A)** and post-drought **(B)** of each species under study. Each point corresponds to a single specimen. Higher CHS indicates healthier trees, while scores of 0 indicate completely defoliated trees (please see also Figure S2). RMSE is the root mean square error of the fitted regression model.

**Table S1.** Percentage loss of conductivity (PLC) and browning score (BS) during drought and post-drought, and percentage of basal area with dead canopies (i.e., browning score = 0 or 1) measured drought and post-drought at each plot. Means ± SE.

| **Geographic area** | **Site** | **Species** | **PLC_drought_, % (n)^1^** | **PLC_post_, % (n)** | **BS_drought_ (n)** | **BS_post_ (n)** | **Dead canopy_drought_ (%)** | **Dead canopy_post_ (%)** |
| --- | --- | --- | --- | --- | --- | --- | --- | --- |
|  |  |  |  |  |  |  |  |  |
| **Northern Table Lands** | Armidale, NSW | *Eucalyptus pauciflora* | 70.8 ± 8.6 (10) | 22.8 ± 5.2 (6)* | 2.21 ± 0.2 (72) | 4.3 ± 0.2 (6) | 54.4 ± 27.2 | 65.4 ± 32.7 |
|  | Mt Duval, NSW | *Eucalyptus dalrympleana* | 49.2 ± 8.7 (10) | 20.9 ± 5.9 (6)* | 2.96 ± 0.1 (109) | 5.0 ± 0.0 (6)* | 31.7 ± 31.6 | 32.0 ± 31.9 |
|  |  | *Eucalyptus laevopinea* | 65.2 ± 6.5 (10) | 11.1 ± 3.2 (5)* | 3.04 ± 0.2 (53) | 4.6 ± 0.2 (5)* | 15.2 ± 4.7 | 16.1 ± 14.2 |
| **Greater Sydney** | Yellomundee, NSW | *Corymbia eximia* | 83.6 ± 6.6 (4) | 20.0 ± 12.3 (5)* | 1.3 ± 0.3 (3) | 5.0 ± 0.0 (3)* | 74.0 | 18.0 |
|  |  | *Eucalyptus crebra* | 84.4 ± 3.3 (4) | 3.9 ± 0.3 (4) | 3.6 ± 0.2 (28) | 5.0 ± 0.0 (4) | 3.1 | 2.1 |
|  |  | *Exocarpos cupressiformis* | - | - | - | - | 12.3 | 11.4 |
| **Central West** | Eugowra Nature Reserve, NSW | *Acacia doratoxylon* | 95.1 ± 3.0 (3) | 13.1 ± 13.1 (5)* | 2.0 ± 0.3 (7) | 5.0 ± 0.0 (5)* | 16.5 ± 4.5 | 21.8 ± 2.1 |
|  |  | *Allocasuarina verticillata* | 86.0 ± 8.2 (3) | 24.6 ± 14.3 (5) | 2.0 ± 0.0 (7) | 3.8 ± 0.5 (5)* | 80.6 ± 19.4 | 23.8 ± 23.8 |
|  |  | *Eucalyptus dealbata* | 90.4 ± 8.1 (4) | 23.4 ± 6.1 (6)* | 1.7 ± 0.2 (6) | 4.7 ± 0.2 (6)* | 20.3 ± 1.3 | 22.0 ± 0.3 |
|  | Peel, NSW | *Eucalyptus macrorhyncha* | 70.5 ± 14.6 (3) | 69.4 ± 6.9 (4) | 1.3 ± 0.3 (3) | 4.3 ± 0.8 (4) | 62.7 ± 23.0 | 54.2 ± 17.7 |
|  |  | *Eucalyptus melliodora* | - | - | - | - | 20.8 ± 7.1 | 15.2 ± 0 |
|  |  | *Eucalyptus polyanthemos* | 70.7 ± 11.5 (4) | 46.7 ± 8.9 (6) | 1.5 ± 0.3 (4) | 4.3 ± 0.2 (6)* | 30.1 ± 8.7 | 12.7 ± 12.7 |
|  | Nangar National Park, NSW | *Acacia doratoxylon* | - | - | - | - | 1.7 | 60.7 |
|  |  | *Eucalyptus albens* | - | - | - | - | 0 | 14.0747541 |
|  |  | *Eucalyptus dealbata* | - | - | - | - | 13.5566003 | 0 |
|  | Mud Hut Road, NSW | *Angophora floribunda* | 63.2 ± 10.6 (4) | 31.0 ± 9.7 (3) | 2.8 ± 0.5 (4) | 4.0 ± 0.6 (3) | 62.8 | 93.3 |
|  |  | *Eucalyptus blakelyi* | 39.7 ± 4.6 (4) | 46.5 ± 13.4 (6) | 3.3 ± 0.3 (4) | 4.3 ± 0.3 (6)* | 50.1 ± 13.3 | 23.1 ± 6.7 |
|  |  | *Eucalyptus melliodora* | - | - | - | - | 0 ± 0 | - |
|  | Henry Bayly, NSW | *Angophora floribunda* | - | - | - | - | 29.1 ± 29.1 | 50.4 ± 41.6 |
|  |  | *Eucalyptus albens* | 19.7 ± 4.0 (5) | 5.2 ± 1.6 (5)* | 3.4 ± 0.2 (5) | 5.0 ± 0.0 (6)* | 6.0 ± 6.0 | 0 ± 0 |
|  |  | *Eucalyptus blakelyi* | - | - | - | - | 0 | 50.8 ± 49.2 |
|  |  | *Eucalyptus macrorhyncha* | 56.8 ± 5.5 (2) | 30.5 ± 7.9 (6) | 2.5 ± 0.5 (2) | 5.0 ± 0.0 (5) | 35.7 ± 35.7 | 35.6 ± 35.6 |
|  | Munghorn Gap National Park, NSW | *Eucalyptus albens* | - | - | - | - | 100 | - |
|  |  | *Eucalyptus blakelyi* | - | - | - | - | 1.1 ± 1.1 | 0 ± 0 |
|  |  | *Eucalyptus macrorhyncha* | - | - | - | - | 0 | 0 |
|  | Billywillinga, NSW | *Eucalyptus albens* | - | - | - | - | 50 ± 50 | - |
|  |  | *Eucalyptus blakelyi* | - | - | - | - | 9.1 ± 9.1 | 11.0 ± 11.0 |
|  |  | *Eucalyptus macrorhyncha* | - | - | - | - | 80.5 ± 7.1 | 64.0 ± 12.2 |
|  |  | *Eucalyptus melliodora* | - | - | - | - | 0 ± 0 | 0 ± 0 |
|  |  | *Eucalyptus polyanthemos* | - | - | - | - | 15.2 ± 3.3 | 24.7 ± 24.7 |
|  | Wattle Flat, NSW | *Eucalyptus albens* | - | - | - | - | 59.1 | 0 |
|  |  | *Eucalyptus macrorhyncha* | - | - | - | - | 58.5 | 35.1 |
|  |  | *Eucalyptus melliodora* | - | - | - | - | 0 | - |
|  |  | *Eucalyptus polyanthemos* | - | - | - | - | 0 ± 0 | 0 ± 0 |
| **Southern Highlands** | Goulburn, NSW | *Eucalyptus macrorhyncha* | 54.0 ± 8.2 (5) | 25.6 ± 9.6 (5) | 2.6 ± 0.2 (5) | 5.0 ± 0.0 (6)* | 49.0 ± 27.4 | 48.3 ± 26.7 |
|  |  | *Eucalyptus mannifera* | - | - | - | - | 40.9 ± 7.0 | 50.0 ± 11.8 |
|  |  | *Eucalyptus rossii* | 59.1 ± 9.1 (4) | 40.5 ± 17.4 (3) | 2.0 ± 0.3 (5) | 3.7 ± 1.3 (3) | 24.9 ±12.5 | 19.2 ± 13.0 |
|  |  | *Exocarpos cupressiformis* | 59.5 ± 9.2 (7) | 40.0 ± 19.8 (4) | - | 4.3 ± 0.8 (4) | 0 | 0 |
|  | Mt Ainslie, ACT | *Allocasuarina verticillata* | 34.0 ± 10.2 (4) | 10.1 ± 3.4 (5)* | - | 5.0 ± 0.0 (5) | 0 ± 0 | 0 ± 0 |
|  |  | *Eucalyptus macrorhyncha* | - | - | - | - | 5.2 | 5.1 |
|  |  | *Eucalyptus mannifera* | 35.5 ± 8.7 (3) | 14.5 ± 7.9 (5) | 2.7 ± 0.6 (6) | 4.1 ± 1.0 (5) | 50.6 ± 26.5 | 55.6 ± 39.2 |
|  |  | *Eucalyptus melliodora* | 66.5 ± 9.4 (7) | 10.0 ± 1.7 (6)* | 3.4 ± 0.2 (7) | 4.7 ± 0.2 (6)* | 0 | 2.1 ± 2.1 |
|  |  | *Eucalyptus rossii* | 79.7 ± 9.5 (4) | 20.5 ± 6.4 (5) | 2.1 ± 0.4 (8) | 2.8 ± 1.2 (5) | 41.5 ± 41.5 | 20.5 ± 20.5 |
|  | Mt Majura, ACT | *Eucalyptus blakelyi* | - | - | - | - | 0 | 14.2 |
|  |  | *Eucalyptus melliodora* | - | - | - | - | 8.3 | 7.4 |
|  | Mt Alexandra, NSW | *Eucalyptus piperita* | 99.9 ± 0.0 (3) | 45.3 ± 8.9 (5) | 1.0 ± 0.0 (2) | 4.2 ± 0.5 (5)* | 51.5 | 35.0 ± 31.8 |
|  |  |  |  |  |  |  |  |  |

^1^ Numbers in brackets indicate the number of trees used per each measurement.

﻿* Asterisks indicate statistically significant differences in the respective parameter during drought and post-drought within a species (P < 0.05).

**Table S2.** Crown attributes and scoring system used in canopy health scores. Each crown attribute is summed, to produce a final canopy health score ranging from 0, for a tree with no leaves remaining, to 25 for a healthy tree. Scoring system modified from Nolan et al. (2021).

| Score | Brief description | Expanded description |
| --- | --- | --- |
| Resprouting |  |  |
| 5 | 0% of resprouts | No visible resprouts |
| 4 | 20-40% | 0–20% of canopy is resprouts in origin |
| 3 | 40-60% | 40–60% of canopy is resprouts in origin |
| 2 | 60-80% | 60–80% of canopy is resprouts in origin |
| 1 | 80-100% | 80–100% of canopy is resprouts in origin |
| 0 | Dead | No canopy |
| Crown size |  |  |
| 5 | Large, vigorous | Well-balanced, fully-extended crown, shaped by large branches containing a healthy 'hierarchy' of smaller branches supporting foliage |
| 3 | Moderate | Moderately-contracted crown, non-uniform in shape with foliage unevenly distributed. Approximately half of the outer, smaller branches dead or missing |
| 1 | Contracted | Contracted Crown contracted, all outer branches dead or missing, foliage on only major branches or stem arising from epicormic growth |
| 0 | Dead | No canopy |
| Dead branches |  |  |
| 5 | 0% | No visible dead branches or branchlets/shoots in the crown |
| 4 | 20-40% | On close inspection some dead terminal branches are evident but not over all the crown |
| 3 | 40-60% | Some small branches are dead but not over all the crown. These are easily observed but do not give the impression of seriously affecting the crown |
| 2 | 60-80% | Some large and or small branches dead over part of the crown with the obvious impression of serious branch death |
| 1 | 80-100% | Large and small branches dead over most of the crown which is obviously dying |
| 0 | Dead | Dead tree |
| Crown density |  |  |
| 5 | Very dense | Very dense leaf clumps with even distribution of clumps over the crown. Very little light penetrating the leaf clumps |
| 4 | Dense | Dense leaf clumps distributed unevenly over the crown |
| 3 | Moderate | Clumps of average density with reasonable distribution or dense clumps very unevenly spread |
| 2 | Sparse | Clumps are sparse and poorly spread |
| 1 | Very sparse | Very few leaves anywhere in crown |
| 0 | Dead | No canopy |
| Canopy browning |  |  |
| 5 | None | No visible discoloration or browning |
| 4 | Minor | 0–10% of canopy exhibits leaf browning and/or discoloration |
| 3 | Moderate | 10–50% of canopy exhibits leaf browning and/or discoloration |
| 2 | Extensive | 50–100% of canopy is discolored, +/- leaf browning |
| 1 | Severe | All of canopy is brown |
| 0 | Dead | No canopy |
